# Electromyography of scapular stabilizers in people without scapular dyskinesis during push-ups: a systematic review and meta-analysis

**DOI:** 10.3389/fphys.2023.1296279

**Published:** 2023-12-05

**Authors:** Ramin Arghadeh, Mohammad Hossein Alizadeh, Hooman Minoonejad, Rahman Sheikhhoseini, Mojtaba Asgari, Thomas Jaitner

**Affiliations:** ^1^ Department of Sports Injury and Biomechanics, Faculty of Sport Sciences and Health, University of Tehran, Tehran, Iran; ^2^ Department of Corrective Exercises and Sports Injury, Faculty of Physical Education and Sport Sciences, Allameh Tabataba’i University, Tehran, Iran; ^3^ Institute for Sport and Sport Science, TU Dortmund University, Dortmund, Germany

**Keywords:** electromyography, scapular, dyskinesis, push-up, unstable surfaces

## Abstract

**Background:** Push-up (PU) is widely considered an effective exercise to stabilize the scapular, especially if performed on unstable surfaces. However, available studies cover a wide range of exercise variations and differ according to exercise prescription, muscle selection and study design. Therefore, findings are contradictory, and conclusions for a proper application of the PU are difficult to draw.

**Objective:** To synthesize the available literature on the changes in the activity of the periscapular muscles in individuals without scapular dyskinesis while performing different types of PU on unstable surfaces.

**Search procedure:** Four online databases were searched from the earliest publications to 9 August 2023, using predefined keywords. Out of the 2,850 potential references identified in the primary search, 92 studies were reviewed in detail, of which 38 met the inclusion criteria and were included. Methodological quality was evaluated using a standardized form based on the Newcastle‒Ottawa scale for observational studies. Data combination was performed using CMA (v3), and the random-effects model was used to calculate the standardized mean difference (SMD) with a 95% confidence interval (CI).

**Results:** The use of unstable surfaces in people without scapular dyskinesis led to increased activity of the upper trapezius during the PU (*p* = 0.017; I^2^ = 84.95%; SMD = 0.425 [95% CI 0.077, 0.773]) and knee PU (*p* = 0.023; I^2^ = 70.23%; SMD = 0.474 [95% CI 0.066, 0.882]) exercises and increased activity of the middle trapezius (MT) (*p* = 0.003; I^2^ = 64.50%; SMD = 0.672 [95% CI 0.225, 1.119]) and serratus anterior (SA) (*p* = 0.039; I^2^ = 4.25%; SMD = 0.216 [95% CI 0.011, 0.420]) muscles during the push-up plus (PUP) exercise.

**Conclusion:** Using an unstable support base during PU does not necessarily increase the activity of all scapular stabilizers. The amount of muscle activity depends on the type of PU other than the type of support base. If an unstable surface is used, PUP exercise appears to be the most effective modality to increase the quality of training, improve performance, and prevent the occurrence of scapular dyskinesis due to the increase in the activity of the MT and SA muscles.

**Systematic Review Registration:** http://www.crd.york.ac.uk/PROSPERO, CRD42021268465.

## 1 Introduction

The optimal function of the scapular is a key component for the appropriate function of the shoulder complex and the proper alignment of the glenohumeral and acromioclavicular joints (Kibler et al., 2012). Mainly, the coordinated activation of the trapezius and serratus anterior (SA) muscles plays an essential role in the motion and stability of the scapular during upper limb movements to support the tightening of the scapular on the thorax as well as the rotations in all three degrees of freedom (Ludewig et al., 2004; Park and Yoo, 2011).

The SA is associated with the normal scapulohumeral rhythm and scapular alignment, and as one of the main upward rotators of the scapular, it enables posterior tilt and scapular protraction (Hwang et al., 2017; Shin et al., 2018). Weakness of this muscle is one of the main reasons for scapular winging, impingement syndrome and shoulder pain (Weon et al., 2011). Additionally, excessive activity of the upper trapezius (UT) or decreased activity of the lower trapezius (LT) and SA may potentially lead to pain, scapular dysfunction, and abnormal scapular movement (Kim et al., 2017). This imbalance of UT and SA muscles in force production can lead to shoulder shrugging due to excessive upward displacement along with inefficient upward rotation and reduction of posterior scapular tilt (Ludewig et al., 2004). Therefore, corrective exercises that intend to restore the function of scapular stabilizer muscles are an important part of rehabilitation programs (Kim et al., 2017).

To identify the most suitable exercises, recruitment patterns of the girdle shoulder muscle during open and closed kinetic chain exercises were studied (Karandikar and Vargas, 2011). Given that open chain exercises cause significant stress on the shoulder joint (Kolber et al., 2010; De Mey et al., 2014), closed chain exercises have become very popular among trainers and therapists and are often included in upper limb rehabilitation (de Araújo et al., 2009). They stimulate proprioception receptors, increase joint congruence, and improve joint dynamic stability through muscle coactivation (Martins et al., 2008). Further, these exercises improve the balance and function of the upper body during daily life and ultimately lead to an increase in self-confidence before return to work or sport (Tucker, 2008; Gioftsos et al., 2016).

The push-up (PU) exercise is one of the preferred closed chain exercises to strengthen scapular stabilizers. It is suggested to perform this exercise on unstable surfaces to increase the involvement of the neuromuscular system and muscle demand needed to maintain postural stability (Ludewig et al., 2004; de Oliveira et al., 2008; Lehman et al., 2008; Andrade et al., 2011; de Araújo et al., 2011; Park and Yoo, 2011). In people with an imbalance of the UT in relation to the SA, the application of exercises aiming to distinctly activate the SA muscle and minimize the activity of the UT (reducing the ratio of the activity of the UT to the SA) simultaneously has been more beneficial than exercises that globally activate several scapulothoracic muscles (Ludewig et al., 2004). For example, the push-up plus (PUP) exercise is one of these exercises that includes full scapular protraction and is usually prescribed to activate and target the scapular stabilizer muscles (Ludewig et al., 2004; Park and Yoo, 2011; San Juan et al., 2015; Torres et al., 2017). Additionally, modifications to the standard PUP, such as PUPs on knees, elbows, walls, and benches, have also been considered mainly in early rehabilitation programs since many people may not be able to perform the standard PUP repeatedly in the initial phases (Ludewig et al., 2004; Park et al., 2014).

The available literature reveals that the rehabilitation of scapular stabilizer muscles is a process that requires the fundamental progress of exercises with an emphasis on increasing the activity of the SA and LT muscles and reducing the activity of the UT muscle simultaneously (Kibler and Sciascia, 2010). Typically, individuals start to train on stable surfaces and then proceed with unstable surfaces induced, e.g., by wobble boards or Swiss balls, in later phases of rehabilitation to increase difficulty and intensity (Lehman et al., 2006). Kang et al. (2019) reviewed the electromyography (EMG) activity of SA and UT muscles during PUP and found that adding an unstable surface increases the activity of the UT but does not affect the activity of the SA (Kang et al., 2019). However, this analysis was limited to one part of the trapezius muscle (upper) and only to one type of exercise (PUP). De Araújo et al. (2021), in another systematic review and meta-analysis, investigated the effect of using unstable exercises on the activity of the periscapular muscles and observed that the EMG activity of the UT and SA increased and decreased, respectively, by adding unstable surfaces. Interestingly, no significant effect was observed on the activity of the middle trapezius (MT) and LT muscles (de Araújo et al., 2021).

De Araújo et al., comprehensively assessed muscle activity during various shoulder girdle and upper limb exercises, including different types of PU and PUP, one-arm and two-arm isometric exercises, shoulder press, inverted row, wall press, bench press, fly, isometric wall press, and isometric bench press. It is important to note that the results presented encompass the entirety of these exercises, and therefore, cannot be solely attributed to PU and PUP exercises.

In a study by Mendez-Rebolledo et al. (2022), muscle activity in the UT and SA muscles was examined during closed kinetic chain exercises on various unstable surfaces (Bosu ball, wobble board, therapeutic ball, and sling). The findings revealed an increasing trend in UT muscle activity on the wobble board, therapeutic ball, and sling compared to stable surfaces. However, none of the unstable tools significantly affected SA activity (Mendez-Rebolledo et al., 2022). Notably, this analysis was limited to the upper part of the trapezius and SA muscles during PU, and the muscle activity in PUP exercises and its variants was not evaluated. Additionally, the study grouped unstable surfaces, exploring their collective impact on the EMG activity of scapular muscles.

To address the gaps in existing literature and considering the significance of all three parts of the trapezius muscle in scapular stabilization and precise movement, we conducted a systematic review and meta-analysis focused exclusively on PU and PUP exercises. Our investigation specifically delves into the effects of using unstable surfaces while performing these exercises on the EMG activity of the trapezius (all three parts) and SA muscles in individuals without scapular dyskinesis. This targeted approach aims to provide a more nuanced understanding of the neuromuscular demands associated with PU and PUP exercises, particularly when performed on unstable surfaces.

## 2 Methods

This study followed the guidelines of the Preferred Reporting Items for Systematic Reviews and Meta-Analyses (PRISMA) and the Cochrane research network (Higgins, 2008; Liberati et al., 2009). The search protocol was preregistered and published in PROSPERO (http://www.crd.york.ac.uk/PROSPERO) with ID code CRD42021268465.

### 2.1 Search strategy

Two blinded members of the research group systematically and independently searched the Web of Science (WOS), PubMed, Scopus, and Google Scholar databases based on the following three main keyword categories described in detail below, without a time limit to start and until 9 August 2023. A crossover search of the eligible references was then performed to ensure a complete census of literature. In addition, the list of references of the final articles included in the research were thoroughly and accurately examined to obtain more information.

1. Scapul* OR shoulder OR glenohumeral OR scapulothoracic OR orientation OR protraction OR malposition OR rhythm OR dysrhythmia OR dyskines* OR dysfunction OR “sick scapul*” OR wing* OR floating OR tipp* OR tilt* OR “scapul* downward rotation syndrome” OR muscle OR muscular
2. Electromyograph* OR “EMG” OR electromyogram OR “root mean square” OR “root-mean-square” OR “RMS” OR pattern OR recruitment OR activ* OR coactiv* OR co-activ* OR cocontract* OR co-contract* OR timing OR onset OR offset
3. Push*-up* OR “push*up*” OR “Push* up*” OR press*-up* OR “press*up*” OR “press* up*” OR “Close* kinetic chain” OR “close* kinematic chain” OR “Close* chain”

### 2.2 Study criteria

Full-text English articles were included if they were published in peer-reviewed journals, reported the mean and standard deviation of the EMG activity of the SA and trapezius muscles or had sufficient indicators to calculate the effect size. Each type of PU had to be performed bilaterally, with the subjects keeping their hands and feet in contact with the support surface during the whole movement.

All review and meta-analysis articles, case reports, and conference articles, which were presented only as abstracts, were excluded from the research.

In addition to the research inclusion criteria, the PICO model was applied to formulate the research question (Eriksen and Frandsen, 2018):

1. Population: Participants who did not have a history of trauma, fracture, surgery, pain or movement limitation in the shoulder joint.
2. Interventions: Different types of PU and PUP exercises on an unstable surface;
3. Comparators: Different types of PU and PUP exercises on a stable surface;
4. Outcomes: EMG activity of the SA and trapezius muscles.

Two independent researchers reviewed all obtained articles. In the first step, after removing duplicates, each of the researchers screened the titles and abstracts separately and retained the articles based on the study criteria. In the second step, each researcher evaluated the eligibility of each article by carefully reading the full texts. Any conflict or difference of opinion regarding the exclusion or inclusion of articles between the two researchers was resolved through discussion and exchange of opinions, or if necessary, by asking the third researcher.

### 2.3 Data extraction

Two researchers independently conducted a detailed and comprehensive review of the preserved articles based on the research inclusion criteria and extracted the following data using a predetermined Excel sheet: 1) name of the first author and year of publication, 2) sex, sample size, and age, 3) type of PU exercise, 4) evaluated muscles, and 5) main findings. It should be noted that if there were unclear data or the published articles were not available, the corresponding author or the first author of the article was contacted through email to receive the missing information or additional explanations.

### 2.4 Methodological quality assessment

The methodological quality of the studies was independently assessed by two researchers using the modified version of the standardized quality assessment form proposed by Siegfried et al. based on the Newcastle‒Ottawa scale (NOS) for observational studies (Siegfried et al., 2005). This tool is recommended in the Cochrane Handbook for systematic review studies and evaluation of various aspects related to internal and external validity of studies (Higgins et al., 2019). The main reason for choosing Siegfried et al.'s form was that instead of presenting a summarized and final score, it provides the possibility of evaluating each of the validity aspects of observational studies separately. In this study, modified versions used in recent systematic reviews on EMG activity of shoulder and scapular muscles during rehabilitation exercises were considered (Ganderton et al., 2013; Schory et al., 2016; Edwards et al., 2017; Kinsella et al., 2017; Karabay et al., 2019; de Araújo et al., 2021).

### 2.5 Statistical analysis

The EMG activity of the scapular stabilizer muscles (mean ± standard deviation) was compared on stable and unstable surfaces. In the studies that reported the standard error of the mean, the standard deviation was calculated using the following formula (Altman and Bland, 2005):

SE = SD/√N (SE = standard error, SD = standard deviation, N = sample size).

For the meta-analysis, the standardized mean difference (SMD) with a 95% confidence interval was calculated (Borenstein et al., 2021). In addition, the random-effects model was used to derive general estimates in all meta-analyses to account for potential heterogeneity among studies. The heterogeneity between studies was calculated using Cochrane’s Q test and I^2^ statistics (Cochran, 1954). The heterogeneity between studies based on the I^2^ statistics was divided as follows by Higgins and Green: low (0%–30%), medium (31%–50%), high (51%–75%), and very high (76%–100%) (Deeks et al., 2019). Furthermore, Begg’s funnel plot, asymmetry test (Egger’s test), and trim-and-fill method were used to evaluate the publication bias of the studies (Egger et al., 1997; Shi and Lin, 2019; Egger et al., 2022). All analyses were performed using CMA software version 3. A *p*-value less than 0.05 was considered statistically significant.

## 3 Results

### 3.1 Study selection

**FIGURE 1:**
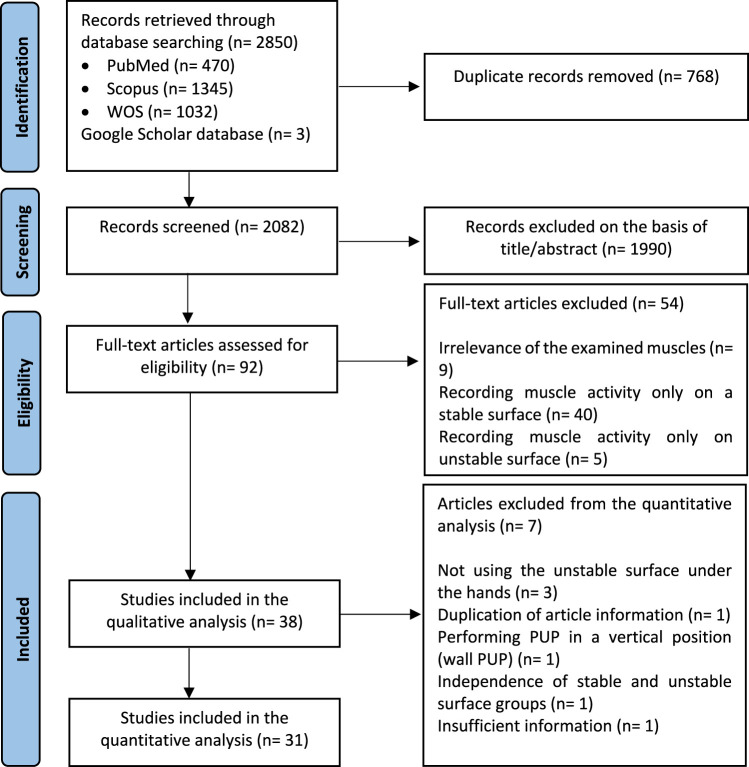
Study flowchart.

Out of the 2,850 records identified in the primary search, 92 full text articles were reviewed in detail to check the eligibility. Thirty-eight studies met the inclusion criteria (Figure 1) and were included in the qualitative analysis (Lear and Gross, 1998; Lehman et al., 2008; Sandhu et al., 2008; Tucker et al., 2008; Maenhout et al., 2010; Park and Yoo, 2011; Tucker et al., 2011; Kim et al., 2012; Park et al., 2013a; Park et al., 2013b; Park et al., 2013c; Lee et al., 2013; Seo et al., 2013; Yoo, 2013; Yoon and Lee, 2013; Calatayud et al., 2014a; Calatayud et al., 2014b; Calatayud et al., 2014c; De Mey et al., 2014; Kim et al., 2014; McGill et al., 2014; Borreani et al., 2015a; Borreani et al., 2015b; Herrington et al., 2015; Lee et al., 2015; Gioftsos et al., 2016; Kim et al., 2016; Harris et al., 2017; Horsak et al., 2017; Torres et al., 2017; de Araújo et al., 2018; Kim and Yoo, 2019; de Araújo et al., 2020; Ferreira et al., 2020; Youdas et al., 2020; De Faria et al., 2021; Patselas et al., 2021; Ratanapinunchai and Madeeyoh, 2022). For the quantitative analysis, 7 studies had to be excluded (Lear and Gross, 1998; Kim et al., 2012; Lee et al., 2013; Yoo, 2013; Yoon and Lee, 2013; Borreani et al., 2015b; Kim et al., 2016).

### 3.2 Characteristics of studies

All the studies included in the research were observational studies that analyzed the EMG activity of the scapular stabilizer muscles in a stable surface compared to an unstable surface. Two of the 38 included studies included two groups (control and scapular dyskinesis) (de Araújo et al., 2018; De Faria et al., 2021)^,^ and the other 36 included only healthy subjects. Twenty-eight out of 38 selected studies were conducted with male subjects (Lehman et al., 2008; Sandhu et al., 2008; Park and Yoo, 2011; Park et al., 2013a; Park et al., 2013b; Park et al., 2013c; Lee et al., 2013; Seo et al., 2013; Yoo, 2013; Yoon and Lee, 2013; Calatayud et al., 2014a; Calatayud et al., 2014b; Calatayud et al., 2014c; Kim et al., 2014; McGill et al., 2014; Borreani et al., 2015a; Borreani et al., 2015b; Lee et al., 2015; Gioftsos et al., 2016; Kim et al., 2016; Torres et al., 2017; de Araújo et al., 2018; Kim and Yoo, 2019; de Araújo et al., 2020; Ferreira et al., 2020; De Faria et al., 2021; Patselas et al., 2021; Ratanapinunchai and Madeeyoh, 2022), 8 studies were conducted with mixed samples (Lear and Gross, 1998; Tucker et al., 2008; Maenhout et al., 2010; Tucker et al., 2011; De Mey et al., 2014; Herrington et al., 2015; Harris et al., 2017; Youdas et al., 2020), and one study was conducted with female subjects (Horsak et al., 2017). Gender was not reported in one study (Kim et al., 2012). The characteristics and main findings of each study are shown in Table 1.

**TABLE 1:** Characteristics of the included studies.

Author	Participants (male/Female)	Age (years)	Intervention(s) (type of PU)	Muscles assessed	Main outcomes
Ratanapinunchai and Madeeyoh (2022)	Healthy = 15 (all male)	21.2 ± 1.01	PUP on stable (bench) and unstable	MSA and LSA	No differences between stable and unstable surfaces
De Faria et al. (2021)	CG = 14 (all male)	CG = 24.57 ± 4.30	PU on stable and unstable (HI, HFI)	SA, UT, MT, and LT	MT activity (CG): unstable > stable
	DG = 13 (all male)	DG = 24.53 ± 2.43			MT activity (unstable): CG > DG
Patselas et al. (2021)	Healthy = 13 (all male)	21.1 ± 1.8	PU and PUP on stable and unstable (compared to elevation exercises)	MSA, LSA, UT, and LT	MSA and LSA activities: PUP (unstable) and PUP (IRP) > elevation exercises
					UT/MSA and UT/LSA ratios: elevation exercises > PU variations
Ferreira et al. (2020)	Healthy = 20 (all male)	22.8 ± 2.5	PUP on stable and unstable (HFI)	MSA, LSA, UT, MT, and LT	LSA activity: unstable > stable
					MSA activity: unstable < stable
De Araújo et al. (2020)	Healthy = 23 (all male)	21.74 ± 3	PUP on stable and unstable (HFI)	MSA, LSA, UT, MT, and LT	MT and LSA activities: unstable > stable
					MSA activity: unstable < stable
Youdas et al. (2020)	Healthy = 32 (22/10)	Male = 24.6 ± 3.2	PU on stable and unstable (HI, FI, HFI)	SA	SA activity: FI > HI, FI > HFI, and stable > HI
		Female = 23.6 ± 1.4			
Kim and Yoo (2019)	Healthy = 11 (all male)	22 ± 1.9	PU and PUP on stable and unstable (NSW)	LT	LT activity (both surfaces): PU phase > PUP phase
De Araújo et al. (2018)	CG = 18 (all male)	CG = 21.50 ± 2.60	PU on stable and unstable (HI)	MSA, LSA, UT, and LT	UT and LSA activities (CG): unstable > stable
	DG = 18 (all male)	DG = 21.89 ± 2.95			MSA and LSA activities (DG): unstable < stable
Harris et al. (2017)	Healthy = 25 (16/9)	27.24 ± 4.02	PU on stable and unstable (sling)	SA and MT	MT activity: unstable > stable
Torres et al. (2017)	Healthy = 20 (all male)	20.9 ± 1.8	PUP on stable and unstable (HFI)	SA, UT, MT, and LT	SA, MT, LT, SA-MT, and UT-LT pairs activities: unstable > stable
Horsak et al. (2017)	Healthy = 19 (all female)	23 ± 3	KPUP and knee plus on stable (bar) and unstable (foam mat, sling)	SA, UT, and LT	activity of all muscles: no differences between stable and unstable surfaces
					UT and LT activities: KPUP > knee-plus
Gioftsos et al. (2016)	Healthy = 13 (all male)	20.5 ± 1.0	PU and PUP on stable and unstable	UT, LT, and SA	UT and LT activities: PU phase > plus phase
					SA activity and UT/LT ratio: PU phase < plus phase
					SA activity: unstable < stable
Kim et al. (2016)	Healthy = 15 (all male)	24.14 ± 0.53	PU on stable and unstable (FI) [FH = 25, 55 cm]	SA	SA activity: no differences between stable and unstable surfaces
Lee et al. (2015)	Healthy = 20 (all male)	24.05 ± 2.21	KPU and KPUP on stable and unstable	UT and SA	UT activity (KPU): Condition 3 > Condition 2 > Condition 1
			Conditions		SA activity (KPUP): Condition 3 > Condition 2 > Condition 1
			1. FH = 0 cm (ground)		
			2. FH = 25 cm		
			3. FH = 30 cm, and HI		
Herrington et al. (2015)	Healthy = 21 (10/11)	22.8 ± 1.4	KPU on stable	SA	SA activity: unstable < stable
			SPU (static) on stable and unstable		
Borreani et al. (2015a)	Healthy = 30 (all male)	23 ± 1.13	PU on stable and unstable (four devices)	SA	SA activity: all unstable surfaces > stable
Borreani et al. (2015b)	Healthy = 29 (all male)	23.5 ± 3.1	PU on stable (HH = 10 and 65 cm) and unstable (HH = 10 and 65 cm)	UT	UT activity: unstable > stable
De Mey et al. (2014)	Healthy = 47 (26/21)	22 ± 4.31	HPU and KPU on stable (bar) and unstable (sling)	SA, UT, MT, and LT	UT and LT activities (HPU): unstable > stable
					SA activity (KPU): unstable < stable
Calatayud et al. (2014a)	Healthy = 29 (all male)	23.5 ± 3.1	PU on stable (HH = 10 and 65 cm) and unstable (HH = 10 and 65 cm)	UT	UT activity: unstable > stable
Calatayud et al. (2014b)	Healthy = 29 (all male)	23.5 ± 3.1	PU on stable and unstable (four types of sling)	UT	UT activity: pulley system > all other types
Calatayud et al. (2014c)	Healthy = 29 (all male)	22.6 ± 2.6	PU on stable and unstable (two types of slings)	SA and UT	SA activity: unstable (both types) < stable
McGill et al. (2014)	Healthy = 14 (all male)	21.1 ± 2.0	PU on stable and unstable (sling)	SA and UT	SA activity: unstable < stable
Kim et al. (2014)	Healthy = 15 (all male)	23.27 ± 1.28	KPUP on stable and unstable (static, oscillating)	SA	unstable (oscillating) > unstable (static) and stable
Yoo (2013)	Healthy = 16 (all male)	26 ± 4.0	WPUP on stable (SFA: 90°, 120°) and unstable (SFA: 90°)	MSA and LSA	MSA activity (SFA: 90°): unstable > stable
					LSA activity: stable (SFA: 120°) > stable (SFA: 90°)
Park et al. (2013a)	Healthy = 12 (all male)	23.7 ± 2.3	PU on stable (with and without hand grips) and unstable (with and without hand grips)	UT and SA	UT activity (with and without hand grips): unstable > stable
					SA activity (without hand grips): unstable > stable
Park et al. (2013b)	Healthy = 16 (all male)	24–26	PU on stable and unstable	UT, LT, and SA	activity of all muscles: unstable > stable
Yoon and Lee (2013)	Healthy = 15 (all male)	22.5 ± 2.16	PUP on stable and unstable (FI)	UT and SA	UT activity: unstable < stable
					SA activity: unstable > stable
Seo et al. (2013)	Healthy = 10 (all male)	24.6	SPUP and KPUP on stable (bench) and unstable (HI)	SA, UT, MT, and LT	MT and LT activities (SPUP): unstable (up phase) > stable
					UT and MT activities (SPUP): unstable (down phase) > stable
					MT activity (KPUP): unstable (up phase) > stable
					SA activity (KPUP): unstable (down phase) > stable
Lee et al. (2013)	Healthy = 20 (all male)	SSG = 23.3 ± 1.45	KPUP on stable (bar) and unstable (sling)	UT, LT, and SA	SA activity: USG (ERP) > SSG
	SSG = 10	USG = 23.7 ± 1.21	Hand position: NP, IRP, and ERP		
	USG = 10				
Park and Yoo (2013C)	Healthy = 14 (all male)	22 ± 2	PU on stable and unstable	UT, LT, MSA, and LSA	UT, LT, and LSA activities: unstable > stable
					MSA and LSA activities: up phase > down phase
Kim et al. (2012)	Healthy = 33 (NR)	21.61 ± 1.32	PU on stable and unstable (FH = 65 cm)	SA	SA activity: foot ball > knee ball, foot ball > knee table
			Conditions		
			1. Foot table		
			2. Knee table		
			3. Foot ball		
			4. Knee ball		
Park and Yoo (2011)	Healthy = 12 (all male)	24.6 ± 2.4	PU and PUP on stable and unstable	MSA, LSA, UT, and LT	MSA and LSA activities (both surfaces): PUP > PU (up phase)
					LSA activity (PUP): unstable > stable
					UT activity (stable): PU (up phase) > PUP
					LT activity and UT/MSA ratio (both surfaces): PU (up phase) > PUP
					UT/LSA ratio (unstable): PU (up phase) > PUP
Tucker et al. (2011)	Healthy = 30 (10/20)	Overhead	PU on stable and unstable (Cuff Link)	SA, UT, MT, and LT	UT, MT, and LT activities: unstable < stable
	Overhead = 15 (5/10)	Male = 21.2 ± 1.3			
	Nonoverhead = 15 (5/10)	Female = 19.5 ± 1.4			
		Nonoverhead			
		Male = 20.2 ± 1.3			
		Female = 19.5 ± 1.2			
Maenhout et al. (2010)	Healthy = 32 (16/16)	22.88 ± 2.43	KPUP on stable and unstable	SA, UT, MT, and LT	SA activity: unstable < stable
					UT/SA ratio: unstable > stable
Sandhu et al. (2008)	Healthy = 30 (all male)	20–30	SPU, KPU, WPU, and EPU on stable and unstable	UT and SA	UT and SA activities: no differences between stable and unstable surfaces
Lehman et al. (2008)	Healthy = 10 (all male)	26.3 ± 1.1	PU and PUP on stable (bench) and unstable (HI, FI)	UT, LT, and SA	activity of all muscles: no differences between stable and unstable surfaces
Tucker et al. (2008)	Healthy = 28 (15/13)	Male = 22.00 ± 3.91	PU on stable and unstable (Cuff Link)	SA, MT, and LT	MT and LT activities: unstable < stable
		Female = 19.69 ± 1.55			
Lear and Gross (1998)	Healthy = 16 (9/7)	Male = 26.9 ± 3.59	PUP on stable and unstable	UT, LT, and SA	SA activity: Conditions 2 or 3 > Condition 1
		Female = 23.9 ± 3.24	Conditions		UT activity: Condition 2 > Condition 1
			1. PUP		
			2. PUP (FH = 45.7 cm)		
			3. PUP (FH = 45.7 cm and HI)		

Abbreviations: NR: not reported, CG: control group, DG: dyskinesis group, SSG: stable surface group, USG: unstable surface group, (S, K, E, W, H) PU: (standard, knee, elbow, wall, half) Push-up, (S, K, W) PUP: (standard, knee, wall) push-up plus, HI: hand instability, FI: feet instability, HFI: hand and feet instability, SFA: shoulder flexion angle, FH: feet height, HH: hands height, NP: neutral position, IRP: internal rotation position, ERP: external rotation position, NSW: narrow shoulder width, (M, L) SA: (Middle, Lower) serratus anterior, UT: upper trapezius, MT: middle trapezius, LT: lower trapezius.

The variety of exercises used in the eligible studies included standard, knee, wall, elbow, and half PUs and standard, knee, wall, and bench PUPs. In addition, various unstable tools, such as oscillating unstable surface, balance board, wobble board, proprioceptive board, balance cushion, balance pads, Airex pad, fitness dome, balance discs, sling, training balls (Bosu ball, Swiss ball, Gym ball, rubber ball, dynamic cushion ball), inflated platforms, foam mat, cuff link, and mini trampoline were used during exercises. According to EMG analysis, the normalization process was performed based on the maximum voluntary isometric contraction (MVIC) (27 studies) (Lear and Gross, 1998; Sandhu et al., 2008; Tucker et al., 2008; Maenhout et al., 2010; Tucker et al., 2011; Lee et al., 2013; Seo et al., 2013; Yoo, 2013; Yoon and Lee, 2013; Calatayud et al., 2014a; Calatayud et al., 2014b; Calatayud et al., 2014c; De Mey et al., 2014; Borreani et al., 2015a; Borreani et al., 2015b; Herrington et al., 2015; Lee et al., 2015; Gioftsos et al., 2016; Kim et al., 2016; Horsak et al., 2017; de Araújo et al., 2018; de Araújo et al., 2020; Ferreira et al., 2020; Youdas et al., 2020; De Faria et al., 2021; Patselas et al., 2021; Ratanapinunchai and Madeeyoh, 2022), the maximal voluntary contraction (MVC) (7 studies) (Lehman et al., 2008; Park et al., 2013a; Park et al., 2013b; Park et al., 2013c; Kim et al., 2014; McGill et al., 2014; Kim and Yoo, 2019), the reference voluntary isometric contraction (RVIC) (1 study) (Torres et al., 2017), the reference voluntary contraction (RVC) (2 studies) (Park and Yoo, 2011; Kim et al., 2012) and the reference isometric contraction (RIC) (1 study) (Harris et al., 2017).

### 3.3 Quality assessment

The quality of the studies was evaluated using the quality assessment form provided by Siegfried et al. based on the NOS (Siegfried et al., 2005). According to the characteristics of the research samples, it may reduce the external validity by reducing the ability to generalize to the general population. Blinding of the examiners while measuring and recording the EMG activity of the muscles was not performed in any of the studies, which increases the risk of bias. However, due to the observational nature of EMG activity analysis, it was not possible to blind the examiners. Since only 7 studies (Seo et al., 2013; Kim et al., 2014; de Araújo et al., 2018; de Araújo et al., 2020; Ferreira et al., 2020; Youdas et al., 2020; De Faria et al., 2021) included a physical examination by one or two clinical experts to evaluate scapular dyskinesis or ensure normal scapulohumeral rhythm and verify the upper limb structures, internal validity in other studies may have been compromised. Six studies (Park et al., 2013a; Yoo, 2013; Yoon and Lee, 2013; Herrington et al., 2015; de Araújo et al., 2020; Ferreira et al., 2020) did not randomize the order of exercises, which increases the risk of selection bias related to potential fatigue. Fourteen studies (Lehman et al., 2008; Maenhout et al., 2010; Kim et al., 2012; Park et al., 2013a; Park et al., 2013b; Yoo, 2013; Herrington et al., 2015; Lee et al., 2015; Kim et al., 2016; Harris et al., 2017; Kim and Yoo, 2019; De Faria et al., 2021; Patselas et al., 2021; Ratanapinunchai and Madeeyoh, 2022) did not include training sessions to familiarize the participants with PU exercises, stable and unstable surfaces, range of motion, body position, and rhythm of PU movements. Moreover, in all studies, except for 14 studies (Lear and Gross, 1998; Lehman et al., 2008; Sandhu et al., 2008; Kim et al., 2012; Lee et al., 2013; Seo et al., 2013; Yoo, 2013; Yoon and Lee, 2013; Kim et al., 2014; Herrington et al., 2015; Lee et al., 2015; Kim et al., 2016; Harris et al., 2017; Ratanapinunchai and Madeeyoh, 2022), exercise techniques were standardized, using the participant’s height to determine the placement of hands and feet or a metronome to control the movement speed of PUs. In all included studies, proper normalization of raw EMG data was performed. However, in only six studies, muscles were randomly selected to record the reference contraction (Harris et al., 2017; Horsak et al., 2017; de Araújo et al., 2018; de Araújo et al., 2020; De Faria et al., 2021; Patselas et al., 2021), which may affect the internal validity of the results (Supplementary Appendix S1).

### 3.4 Qualitative analysis

The total sample included in the review was 826 (126 women, 667 men and 33 people of unknown sex), of whom 31 were men with scapular dyskinesis and the rest were healthy humans. Closed chain exercises cover standard, knee, wall, elbow, and half PUs as well as standard, knee, wall, and bench PUPs. Activities of UT (27 studies) (Lear and Gross, 1998; Lehman et al., 2008; Sandhu et al., 2008; Maenhout et al., 2010; Park and Yoo, 2011; Tucker et al., 2011; Park et al., 2013a; Park et al., 2013b; Park et al., 2013c; Lee et al., 2013; Seo et al., 2013; Yoon and Lee, 2013; Calatayud et al., 2014a; Calatayud et al., 2014b; Calatayud et al., 2014c; De Mey et al., 2014; McGill et al., 2014; Borreani et al., 2015b; Lee et al., 2015; Gioftsos et al., 2016; Horsak et al., 2017; Torres et al., 2017; de Araújo et al., 2018; de Araújo et al., 2020; Ferreira et al., 2020; De Faria et al., 2021; Patselas et al., 2021), MT (10 studies) (Tucker et al., 2008; Maenhout et al., 2010; Tucker et al., 2011; Seo et al., 2013; De Mey et al., 2014; Harris et al., 2017; Torres et al., 2017; de Araújo et al., 2020; Ferreira et al., 2020; De Faria et al., 2021), LT (20 studies) (Lear and Gross, 1998; Lehman et al., 2008; Tucker et al., 2008; Maenhout et al., 2010; Park and Yoo, 2011; Tucker et al., 2011; Park et al., 2013b; Park et al., 2013c; Lee et al., 2013; Seo et al., 2013; De Mey et al., 2014; Gioftsos et al., 2016; Horsak et al., 2017; Torres et al., 2017; de Araújo et al., 2018; Kim and Yoo, 2019; de Araújo et al., 2020; Ferreira et al., 2020; De Faria et al., 2021; Patselas et al., 2021) and SA (34 studies) (Lear and Gross, 1998; Lehman et al., 2008; Sandhu et al., 2008; Tucker et al., 2008; Maenhout et al., 2010; Park and Yoo, 2011; Tucker et al., 2011; Kim et al., 2012; Park et al., 2013a; Park et al., 2013b; Park et al., 2013c; Lee et al., 2013; Seo et al., 2013; Yoo, 2013; Yoon and Lee, 2013; Calatayud et al., 2014b; De Mey et al., 2014; Kim et al., 2014; McGill et al., 2014; Borreani et al., 2015a; Herrington et al., 2015; Lee et al., 2015; Gioftsos et al., 2016; Kim et al., 2016; Harris et al., 2017; Horsak et al., 2017; Torres et al., 2017; de Araújo et al., 2018; de Araújo et al., 2020; Ferreira et al., 2020; Youdas et al., 2020; De Faria et al., 2021; Patselas et al., 2021; Ratanapinunchai and Madeeyoh, 2022) were evaluated.

### 3.5 Quantitative analysis

To determine the effect of unstable surfaces on the activity of scapular stabilizer muscles, studies were grouped based on exercises and muscles. A random-effects model was used in all meta-analyses to reduce the possible effect of data heterogeneity on the research results.

**FIGURE 2:**
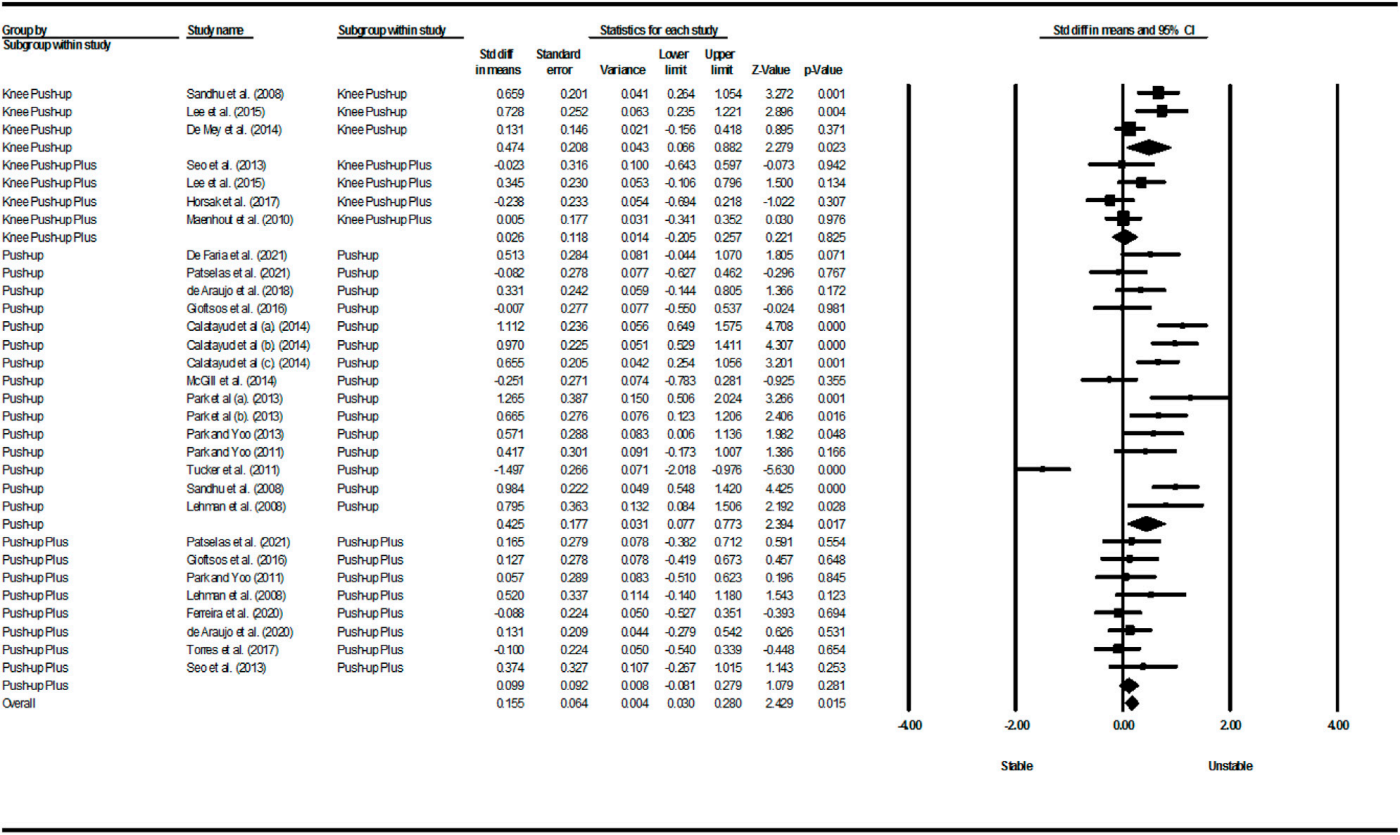
Forest plot of the UT muscle EMG activity.

**FIGURE 3:**
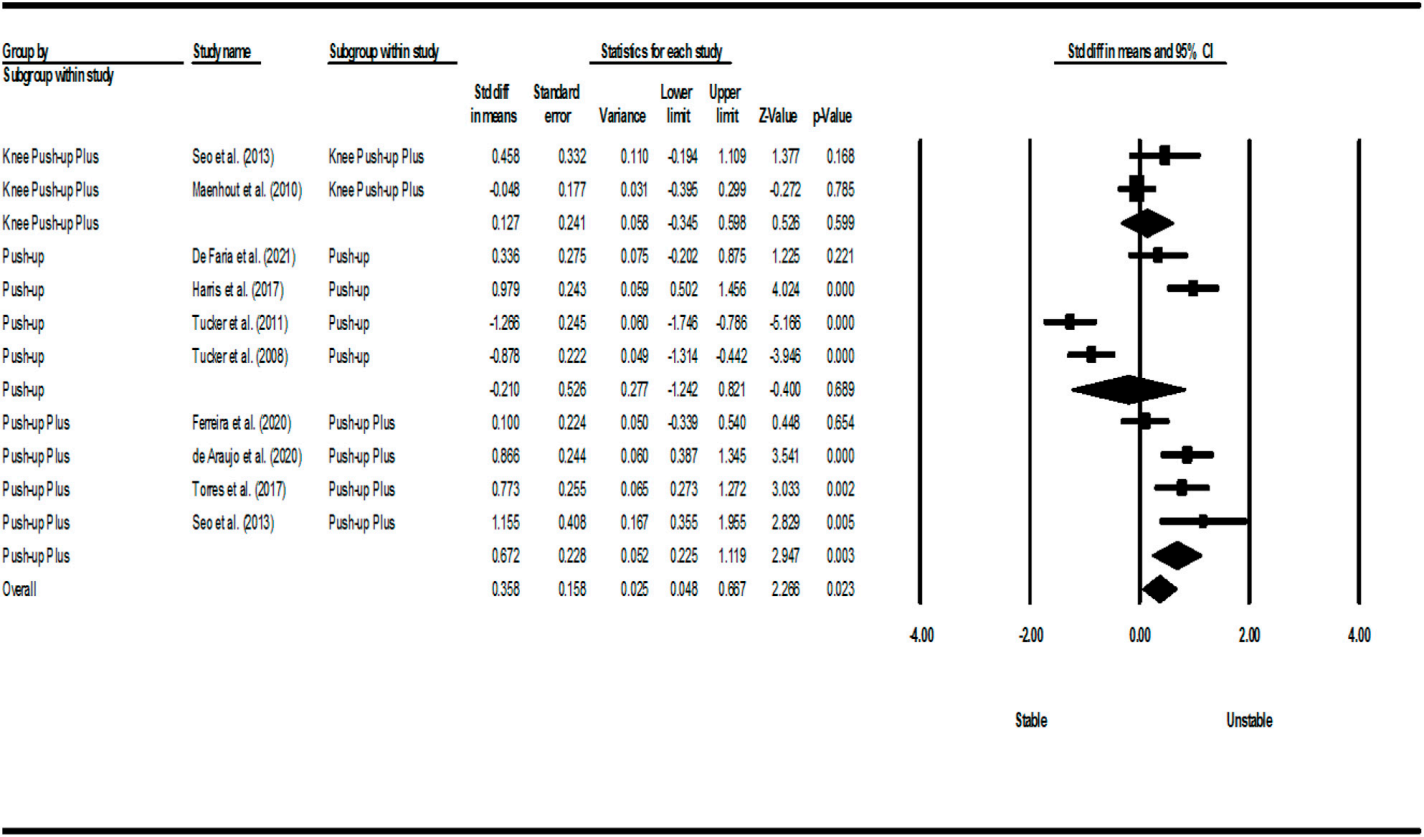
Forest plot of the MT muscle EMG activity.

**FIGURE 4:**
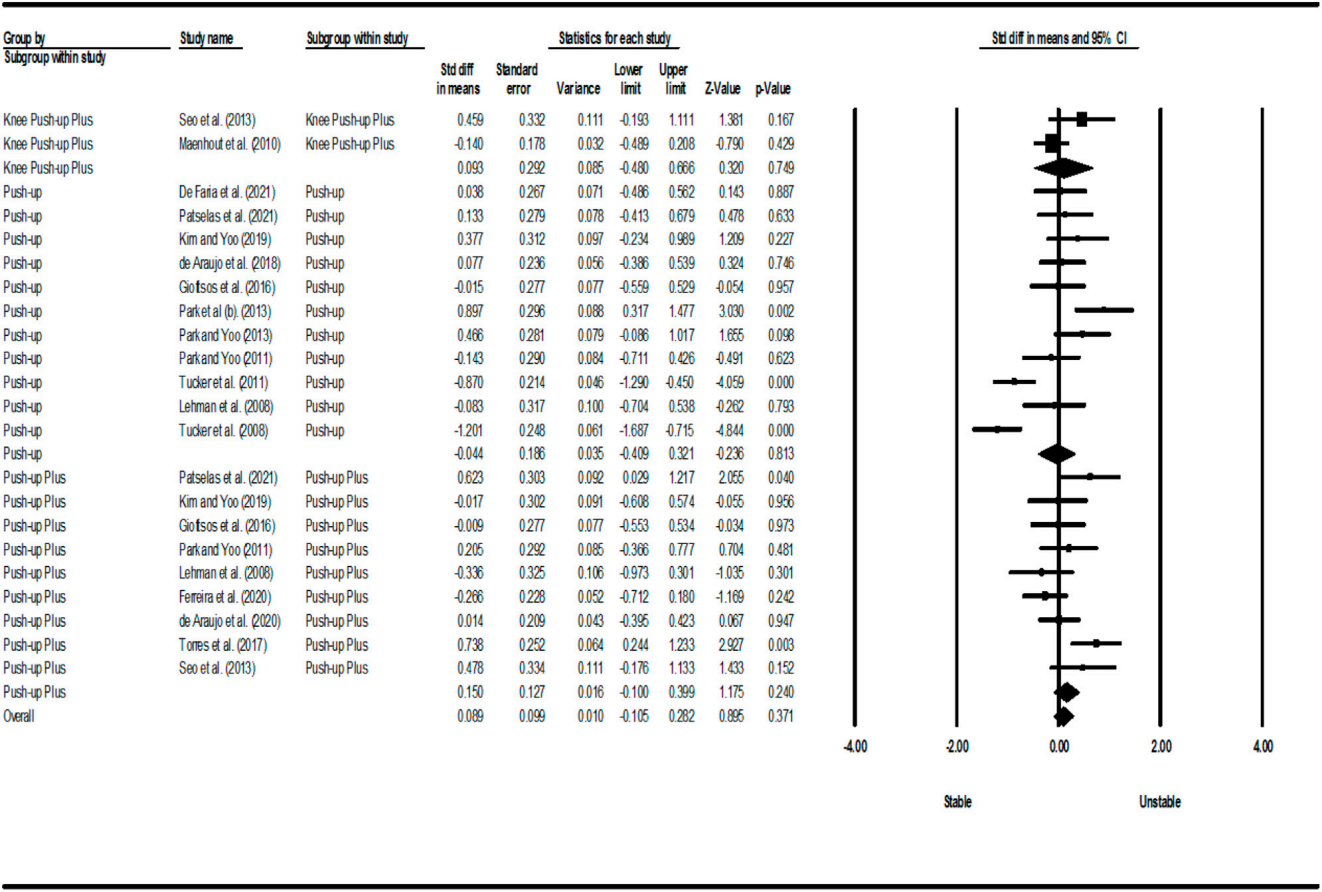
Forest plot of the LT muscle EMG activity.

**FIGURE 5:**
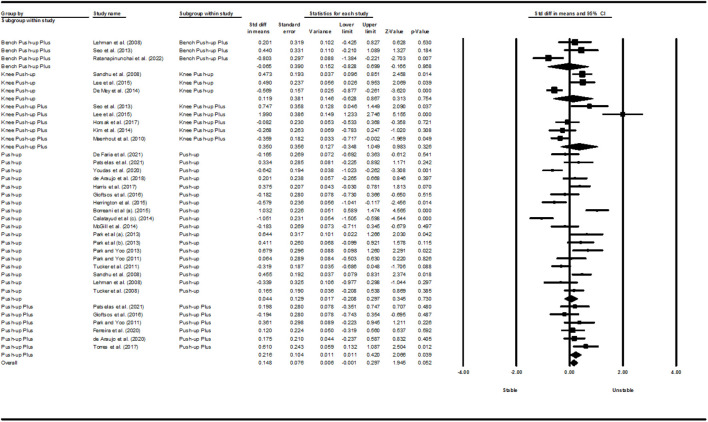
Forest plot of the SA muscle EMG activity.

Figures 2–5 show the results of the activity of the trapezius (three parts) and SA muscles in different types of PU. Meta-analysis of exercise subgroups showed that there was no significant difference between stable and unstable surfaces during PUP (*p* = 0.281; I^2^ = 0%) and knee PUP (*p* = 0.825; I^2^ = 7.60%) for the UT; PU (*p* = 0.689; I^2^ = 94.56%) and knee PUP (*p* = 0.599; I^2^ = 44.62%) for the MT; PU (*p* = 0.813; I^2^ = 80.75%), PUP (*p* = 0.240; I^2^ = 48.37%), and knee PUP (*p* = 0.749; I^2^ = 60.44%) for the LT; or PU (*p* = 0.730; I^2^ = 80.50%), knee PU (*p* = 0.754; I^2^ = 91.45%), knee PUP (*p* = 0.326; I^2^ = 88.85%), and bench PUP (*p* = 0.868; I^2^ = 78.12%) for the SA. On the other hand, adding an unstable surface led to an increase in the activity of the UT during PU (*p* = 0.017; I^2^ = 84.95%; SMD = 0.425 [95% CI 0.077, 0.773]) and knee PU (*p* = 0.023; I^2^ = 70.23%; SMD = 0.474 [95% CI 0.066, 0.882]); the MT during PUP (*p* = 0.003; I^2^ = 64.50%; SMD = 0.672 [95% CI 0.225, 1.119]); and the SA during PUP (*p* = 0.039; I^2^ = 4.25%; SMD = 0.216 [95% CI 0.011, 0.420]).

**FIGURE 6:**
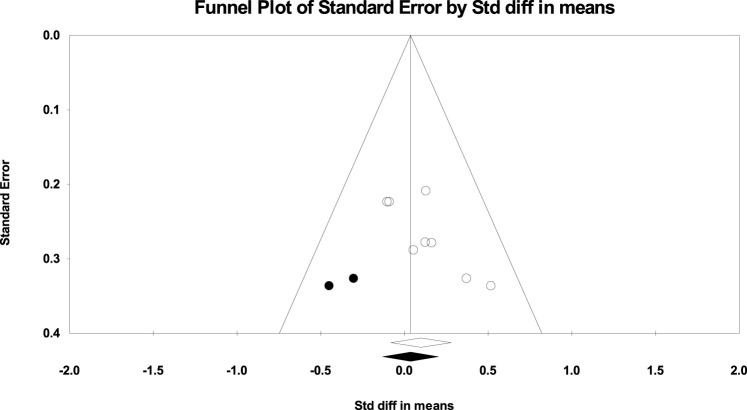
Funnel plot of the UT muscle (Push-up Plus).

**FIGURE 7:**
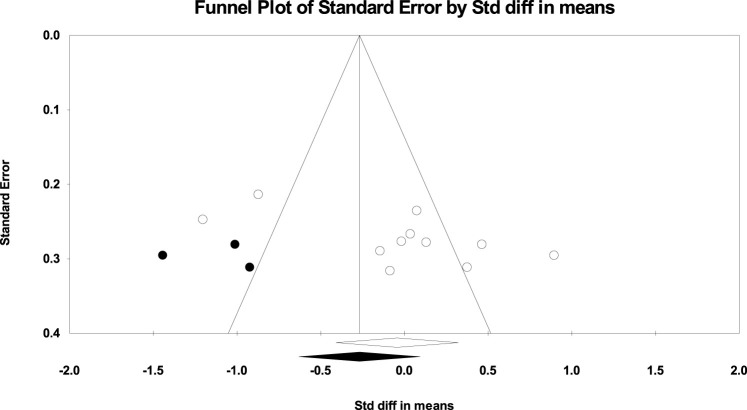
Funnel plot of the LT muscle (Push-up).

**FIGURE 8:**
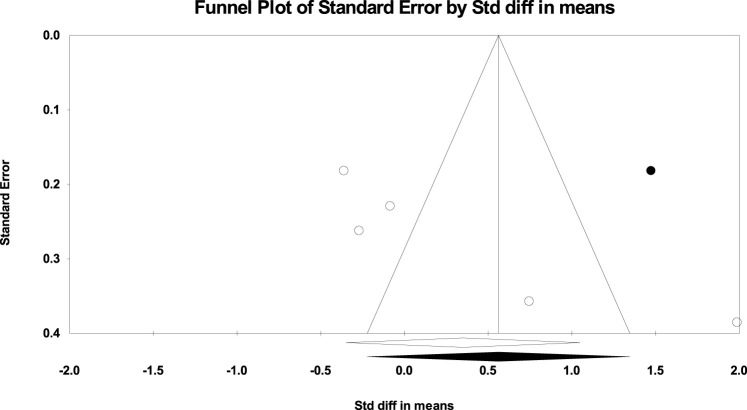
Funnel plot of the SA muscle (Knee Push-up Plus).

The absence of publication bias was confirmed using Egger’s test for the UT in PU (*p* = 0.665), knee PU (*p* = 0.215), and knee PUP (*p* = 0.973) studies; for the MT in PU (*p* = 0.565) and PUP (*p* = 0.342) studies; for the LT in PUP (*p* = 0.565) studies; and for the SA in PU (*p* = 0.615), PUP (*p* = 0.909), knee PU (*p* = 0.333), and bench PUP (*p* = 0.099) studies. However, according to the significance level of Egger’s test for the UT in PUP (*p* = 0.027) studies, for the LT in PU (*p* = 0.029) studies, and for the SA in knee PUP (*p* = 0.046) studies, publication bias was observed, as shown in Figures 6–8.

## 4 Discussion

The aim of the current systematic review was to analyze the effects of using unstable surfaces during PU and PUP exercises on the EMG activity of the scapular stabilizer muscles in people without scapular dyskinesis. The findings demonstrate that using an unstable support base does not necessarily increase the activity of all scapular stabilizer muscles. In detail, the amount of muscle activity depends on both the type of support base and the type of PU exercise. Given the extent of the research findings, the EMG activity of each muscle during different types of PU and PUP exercises is discussed separately.

### 4.1 Trapezius muscle

An increase in the activity of the UT muscle during PU and knee PU as well as an increase in the activity of the MT muscle during PUP on unstable surfaces compared to stable surfaces in subjects without scapular dyskinesis was observed. However, performing on unstable surfaces did not show a significant effect on the EMG activity of the middle and lower parts of the trapezius muscle during the PU exercise, the upper and lower parts of the trapezius muscle during the PUP exercise, or all three parts of the trapezius muscle during the knee PUP exercise. Such conflicting results might be explained by different methodological approaches among the studies.

The increase in the activity of the UT muscle during the PU and knee PU exercise on the unstable surface is probably due to the synergistic role of this muscle in neutralizing unnecessary movements needed to stabilize the scapular (Lear and Gross, 1998; Calatayud et al., 2014a; Calatayud et al., 2014c). In other words, placing the hands on unstable surfaces during PU causes excessive disturbances, vibrations, and shoulder elevation. As a result, increased activity of the UT neutralizes such unconscious movements. Furthermore, the inefficacy of unstable surfaces on the activity of the UT muscle during PUP and knee PUP exercises may be due to the compensatory neuromuscular control mechanisms of other shoulder muscles (Sandhu et al., 2008). However, the addition of the “plus phase” to different types of PU exercises appears to be the main reason for the differences between the studies. In fact, it can be concluded that adding a “plus phase” when using unstable surfaces may be a suitable solution to prevent an increase in UT muscle activity (Lehman et al., 2008; Horsak et al., 2017; Torres et al., 2017; de Araújo et al., 2020; Ferreira et al., 2020). Horsak et al. (2017) emphasized that compared to the knee plus, the knee PUP activates the upper and lower parts of the trapezius muscle (Horsak et al., 2017). The additional flexion and extension of the elbow and the subsequent increase in physical demands to stabilize the shoulder complex may be the reason for the slight increase in the activity of the upper and lower parts of the trapezius muscle during knee PUP compared to knee plus (Horsak et al., 2017). Hence, it appears that the emphasis is placed on the knee plus exercise as a priority over both the standard PUP and knee PUP exercises. This approach aims to reduce UT muscle activity among individuals engaged in overhead sports, with the goal of mitigating scapular dyskinesis. Additionally, this exercise may be suitable for correcting scapular dyskinesis related to muscle imbalance, especially in the initial phases of rehabilitation programs.

Our findings align with the outcomes of systematic reviews conducted by De Araújo et al. (2021) and Mendez-Rebolledo et al. (2022), indicating an overall increase in UT muscle activity (de Araújo et al., 2021; Mendez-Rebolledo et al., 2022). However, our results concerning the “plus” phase of PUP and knee PUP exercises, specifically the absence of a significant effect of the unstable surface on UT activity, differ from the conclusions drawn in the systematic review by Kang et al. (2019) (Kang et al., 2019).

Kang et al. (2019) reported a 2.85% MVIC increase in UT activity when an unstable surface was introduced during PUP exercises. It is noteworthy that this increase in activity can be attributed to the inclusion of subjects with scapular dyskinesis in the studies analyzed by Kang et al. (2019). In contrast, our study exclusively focused on healthy subjects without scapular dyskinesis. This divergence in subject characteristics may contribute to the variance in outcomes between our study and that of Kang et al. (2019).

Of particular interest is the meta-analysis conducted by Kang et al. (2019), where the most substantial mean difference for the UT muscle was observed in the study by Pirauá et al. (2014). Notably, Pirauá et al. (2014) included subjects with scapular dyskinesis, further highlighting the potential impact of differing subject populations on UT muscle activity outcomes during PUP exercises.

Regarding the middle and lower parts of the trapezius, the use of an unstable surface in any of the PU types (except for the increase in the MT activity during the PUP) showed no significant effect on the EMG activity. Maeo et al. (2014) studied muscular activities during PU exercise in static and dynamic conditions on unstable (sling) and stable (ground) surfaces and observed that in the static condition on the sling, the percentage of maximum EMG values of the biceps brachii and triceps brachii muscles is significantly higher than that on the ground. Under dynamic conditions, such a difference was also significant in the pectoralis major muscle in addition to the biceps brachii and triceps brachii muscles (Maeo et al., 2014). In a similar study, De Mey et al. (2014) evaluated the activity levels of shoulder muscles during knee PU and half PU exercises on stable and unstable (sling) surfaces and found a decrease in the activity of the scapular muscles and an increase in the activity of the glenohumeral muscles during sling exercises (De Mey et al., 2014). These findings support the argument recently raised by Horsak et al. (2017) that the global stabilizers of the shoulder girdle play an important role in stabilizing the glenohumeral joint on unstable surfaces; therefore, there is no need to significantly increase the activity of the periscapular muscles (Horsak et al., 2017). Hence, it seems that unstable surfaces do not induce significant disturbances in the scapular that require higher neuromuscular demands of these muscles during PUs.

The increase in the activity of the MT muscle during the PUP exercise on unstable surfaces might be explained by external factors such as the location and the type of the unstable surface. De Araújo et al. (2020) and Torres et al. (2017), for example, placed unstable surfaces under the hands and feet (double instability) (Torres et al., 2017; de Araújo et al., 2020). Therefore, the degree of instability applied to the entire kinetic chain probably not only generates a greater need for neuromuscular control and balance in the upper limbs but may also involve the anterior trunk muscles (abdominal muscles). In other words, the increase in the activity of the anterior trunk muscles may occur due to the need for greater stability of the trunk due to double instability, leading to stronger muscle contractions in the abdominal area due to the prone position of the body during the PUP exercise (Vera-Garcia et al., 2000; Behm and Anderson, 2006; Maeo et al., 2014; de Souza Bezerra et al., 2020).

As the location of the unstable surface and the type of unstable surface in the study of Ferreira et al. (2020) were similar to those in De Araújo et al. (2020) and Torres et al. (2017), an increase in the activity of the MT muscle was expected. However, the use of unstable surfaces in this study had no effect on the EMG activity of the trapezius muscle (Ferreira et al., 2020). The main difference between this study and the other two studies is the way the PUP exercise is performed. PUP was performed isometrically in the study of Ferreira et al. (2020) and dynamically in the studies of De Araújo et al. (2020) and Torres et al. (2017). Thus, it seems that the position adopted during isometric exercises puts the scapular in a position where there is no need for significant activity of the trapezius muscle.

Unlike the aforementioned studies, Seo et al. (2013) reported superior activity of the MT muscle, although the unstable surface was placed only under the hands (single instability). The increase in the activity of the MT muscle was probably due to the type of stable and unstable surface used in this study (Seo et al., 2013). The three studies mentioned above used the ground and the Bosu ball as stable and unstable surfaces, respectively, while Seo et al. (2013) applied a chair and a Swiss ball as stable and unstable surfaces, respectively.

The overall result of our meta-analysis for the MT muscle (increased activity) is inconsistent with the result of a recent review published by De Araújo et al. (2021) (no significant effect of the unstable surface on the MT activity) (de Araújo et al., 2021). The main reason for the inconsistent results can be found in the “two-arm isometric” exercise subgroup in the review by De Araújo et al. (2021). This subgroup includes studies that have either not been published in English (Batista et al., 2013) or have evaluated the effect of unstable surfaces on the MT activity in the plank exercise (Biscarini et al., 2019). Interestingly, the result of the meta-analysis of this subgroup showed that there is no significant difference between stable and unstable surfaces (*p* = 0.38). In fact, the meta-analysis result of the “two-arm isometric” exercise subgroup has influenced the final meta-analysis result of the MT muscle (*p* = 0.10) in this study. It is important to note that our review includes studies that focused only on different types of PU and PUP exercises and were published in English.

### 4.2 SA muscle

For the SA muscle, the PUP exercise on unstable surfaces leads to an increase in muscle activity in people without scapular dyskinesis. However, there was no significant effect on the EMG activity of the SA during the PU, knee PU, knee PUP, and bench PUP exercises. The location of the electrodes, the location of the unstable surfaces, the feet height, the type of unstable surfaces, variations in exercise performance, and the normalization method of the EMG signals are factors that might help explain heterogeneity of the literature.

In the studies where no significant differences were observed, the electrodes were placed on the SA-fifth fibers (middle SA) (Lehman et al., 2008; Park et al., 2013c; de Araújo et al., 2018; Ratanapinunchai and Madeeyoh, 2022). Conversely, in studies that reported increased EMG activity on an unstable surface, the electrodes were positioned on the SA-seventh fibers (lower SA) (Park and Yoo, 2011; Park et al., 2013a; Park et al., 2013b; Park et al., 2013c; Seo et al., 2013; Kim et al., 2014; Borreani et al., 2015a; Lee et al., 2015; de Araújo et al., 2018; de Araújo et al., 2020; Ferreira et al., 2020). Notably, while Yoo (2013), Ferreira et al. (2020), and De Araújo et al. (2020) evaluated the activity of the middle SA, Yoo’s study (2013) showed increased activity during the wall PUP exercise (Yoo, 2013). On the other hand, Ferreira et al. (2020) and De Araújo et al. (2020) found a decrease in middle SA muscle activity due to excessive instability (hands and feet) when exposed to an unstable surface (Ferreira et al., 2020, De Araújo et al., 2020).

Some researchers believe that high levels of instability may cause problems in muscle recruitment and thus reduce EMG activity (Calatayud et al., 2014b; De Mey et al., 2014; Behm et al., 2015). The literature findings show that increasing instability during a task or exercise has a negative effect on the EMG amplitude and force output (Anderson and Behm, 2005). On the other hand, Ratanapinunchai and Madeeyoh (2022) and Herrington et al. (2015) observed no difference and decreased activity of the SA muscle when using unstable surfaces, respectively, despite measuring the activity of SA-seventh fibers. The different EMG responses of the lower SA muscle to the addition of an unstable surface in these two studies may be due to the bench PUP in the study of Ratanapinunchai and Madeeyoh (2022) and the static nature of the PU in the study of Herrington et al. (2015) (Herrington et al., 2015; Ratanapinunchai and Madeeyoh, 2022). The meta-analysis results of our research strengthen the theoretical hypothesis first proposed by Park and Yoo (2011). They evaluated the activity of different parts of the SA during PUs on stable and unstable surfaces and suggested that the lower SA plays a more important role than the middle SA in maintaining the scapular position under unstable conditions; thus, the neuromuscular demand of this part is higher (Park and Yoo, 2011). Additionally, some studies have shown that performing PU with an unstable tool (under the hands or under the legs) can increase the EMG activity of the abdominal muscles (Freeman et al., 2006; Lehman et al., 2006; Beach et al., 2008; Calatayud et al., 2014c; Maeo et al., 2014; de Souza Bezerra et al., 2020). These findings are also confirmed by Behm and Colado (2012), who indicated the existence of a consensus regarding the positive effect of unstable surfaces in increasing the neuromuscular demand of the axial muscles (Behm and Colado, 2012). Therefore, due to the existence of an anatomical-functional relationship between the abdominal oblique muscles (especially the external oblique) and the SA (especially the lower part), the use of two strategies of conscious contraction of abdominal muscles and an unstable surface at the same time during PU might lead to an increase in the lower SA (Myers, 2013; Toro et al., 2016; de Araújo et al., 2020; Ferreira et al., 2020). Indeed, a combination of strategies may be useful when the clinical goal is to improve scapular stability. These findings provide new evidence and strengthen the theories of force transmission along the kinetic chain and anatomical pathways (McMullen and Uhl, 2000; Maenhout et al., 2010). Therefore, it can be concluded that people participating in overhead sports are exposed to scapular dyskinesis over time due to the repetitive nature of their movements (kinesiopathological model) (Sahrmann et al., 2017)^,^ and there may be a disturbance in the transmission of force between the trunk and the scapular and possibly the upper limb due to inappropriate activation or strength weakness of the external oblique muscle. Hence, muscle activity and function of the SA might also be affected negatively. Such a functional relationship between the SA and external oblique muscles supports the theoretical arguments presented about the correction of scapular dyskinesis related to muscle imbalance, whereby core exercises are recommended.

One of the important factors in the studies that reported the decrease in the activity of the SA muscle on the unstable surface might be an insufficient adjustment of the leg height after the addition of the unstable surface to maintain the alignment of the trunk (Maenhout et al., 2010; De Mey et al., 2014; McGill et al., 2014; Herrington et al., 2015; Youdas et al., 2020). The decrease in the activity of the SA muscle on the unstable surface in this type of study is probably due to the higher position of the hands that places more or less weight on the lower limb and the upper limb, respectively. Similarly, Lehman et al. (2006) showed that by raising the legs during the standard PUP, more weight is placed on the upper limb, and SA activity increases (Lehman et al., 2006). McGill et al. (2014) also revealed that the surface on which the PU is performed may have less effect than differences in exercise performance and suggested that the SA muscle is preferentially activated by exercises in which the line of action is in the same direction as gravity. In other words, straight pushing from the chest activates the SA more than angular pushing (McGill et al., 2014).

The type of unstable surface, the method of performing the exercise and the method of normalizing the EMG signals seem to be the confounding factors in studies that did not report any difference in the activity of the SA muscle on the unstable surface. The tool used to create instability in the study of De Faria et al. (2021) exclusively caused internal-external instability, which may not have created enough challenge for the neuromuscular system to increase SA activity (De Faria et al., 2021). Horsak et al. (2017) and Kim et al. (2014) used foam mats and dynamic cushion balls as unstable surfaces, respectively. Since the unstable surfaces used in these studies might not induce enough instability, there was no need for maximum contraction of SA (Kim et al., 2014; Horsak et al., 2017). Tucker et al. (2008) also used the cuff link device, which is a tool used in rehabilitation to stimulate the closed kinetic chain of the upper limb. Although the activity of the SA was slightly higher when using the cuff link, the activity levels of this muscle during the standard PU and cuff link were similar. Therefore, if the goal is to activate the SA and the person does not have enough upper body strength to perform a standard PU, a cuff link seems to be a suitable alternative. Nonetheless, if there is a need for higher levels of activity of the MT and LT muscles and SA, the standard PU is a more appropriate exercise (Tucker et al., 2008). Additionally, in some studies that did not report any difference in the activity of the SA muscle on the unstable surface, the exercise was performed isometrically (de Oliveira et al., 2008; Sandhu et al., 2008; de Araújo et al., 2011). Harris et al. (2017) used RIC instead of MVIC to normalize the signals (Harris et al., 2017). The different normalization process of the signals in this study compared to other studies might be the reason for the inefficacy of the unstable surface on the activity of the SA muscle.

Considering the results of the meta-analysis concerning the SA muscle in the context of the PUP exercise, it becomes evident that distinct exercise phases necessitate the engagement of various muscles exhibiting varying degrees of activity. These variations arise from differing movements and ranges of motion (Park and Yoo, 2011). The PU phase mainly includes arm elevation along with scapular movement due to the activity of the UT and LT muscles. In contrast, the plus phase only involves scapular movement, which mainly leads to SA activity. Therefore, the PUP exercise has been the most preferred to increase the activity of the SA muscle. However, it should be noted that PUP is a difficult exercise that requires the activity of the whole body; it is difficult to monitor and, consequently, perform it correctly (Gioftsos et al., 2016). To solve this problem, modified PUP exercises such as knee PUP and bench PUP are recommended (Ludewig et al., 2004; Lehman et al., 2008; Park et al., 2014; Ratanapinunchai and Madeeyoh, 2022). However, the results of the meta-analysis showed that the use of an unstable surface during knee PUP and bench PUP does not have a significant effect on the activity of the SA. The lack of influence of the unstable surface on the activity of this muscle during modified PUP exercises can be attributed to the body position. Considering the starting position of the knee PUP (distal point of the knee on the ground) compared to common exercises performed on the hands and feet, as well as applying more load to the lower limb due to the body slope caused by placing the hands on the bench in the bench PUP, less load is imposed on the scapulothoracic joint (Lehman et al., 2008; Kim et al., 2014). Therefore, the maximum contraction of the SA was not needed. Although PUP is considered a more effective form of exercise to activate SA than standard PU, caution should be exercised when using this exercise in sports or clinical settings. Lunden et al. (2010) reported that scapulothoracic and glenohumeral movement in PUP may reduce the subacromial space and lead to impingement of the arm rotator muscles (Lunden et al., 2010).

The overall impact of introducing an unstable surface on the EMG activity of the SA muscle in our study, denoted by the absence of a significant effect, aligns with recent reviews by Kang et al. (2019) and Mendez-Rebolledo et al. (2022), yet contrasts with the findings of the systematic review by De Araújo et al. (2021) (Kang et al., 2019; de Araújo et al., 2021; Mendez-Rebolledo et al., 2022).

The discrepancy in results with De Araújo et al. (2021) can be attributed to the “one-arm isometric” exercise subgroup within their study, which primarily contributes to the observed difference. This subgroup includes studies assessing the impact of unstable surfaces on SA activity during unilateral exercises. Specifically, the reduction in SA activity induced by axial load exercises in this subgroup (*p* = 0.010) significantly influenced the final meta-analysis result for SA activity, indicating a decrease (*p* = 0.008).

It is crucial to note that our study exclusively incorporates investigations where each type of PU was executed bilaterally, providing a more focused examination of the effects of unstable surfaces on SA muscle activity during PU and PUP exercises.

### 4.3 Practical relevance

Our study elucidates the biomechanical demands associated with various PU and PUP exercises performed on unstable surfaces, specifically concerning the activity levels of scapular stabilizer muscles. This information holds practical significance for athletes, coaches, and therapists, enabling them to make informed decisions when selecting the most appropriate type of PU or PUP based on their training objectives.

By tailoring PU variations according to the reported muscle activity in different parts of the trapezius and the SA, individuals can progressively enhance upper limb control, mitigating the risk of scapular dyskinesis stemming from muscle imbalances over the long term.

## 5 Conclusion

Using an unstable support base does not necessarily increase the activity of all scapular stabilizer muscles. The amount of muscle activity depends on both the type of support base and the type of PU exercise. Therefore, the results of this review provide a basis for the guidance and selection of appropriate exercise programs for therapists and other sports professionals. It allows us to prescribe how different types of PUs stimulate specific muscles to prevent muscle imbalance and finally the occurrence of scapular dyskinesis, especially in people participating in overhead sports.

## 6 Limitations

Although the quality of the analyzed studies was high, our study has the following limitations: our results are limited to healthy and asymptomatic scapulars. Therefore, the obtained results cannot be generalized for people with shoulder or scapular dysfunction with pain, such as subacromial impingement syndrome. The PU phase, in which the EMG activity was recorded, was either not the same in all studies or was not reported at all, which could affect the results of the study.
