# Supplementary material for: Electromyography of scapular stabilizers in people without scapular dyskinesis during push-ups: a systematic review and meta-analysis
Source: Front Physiol. 2023 Dec 5;14:1296279. doi: 10.3389/fphys.2023.1296279 (PMC10728295; doi:10.3389/fphys.2023.1296279)
Supplement: Supplementary file 1 [file Table1.DOCX]

| **Appendix 1.** Quality Assessment of Included Studies | | | | | | | | | | | |
| --- | --- | --- | --- | --- | --- | --- | --- | --- | --- | --- | --- |
| **Study** | **External validity** | | **Internal validity** | | | | | | | | |
|  |  |  | **Performance** | **Detection** | | **Selection bias and control of confounding** | | | | | |
| **Author** | **Representative sample** | **Participation rate** | **Direct observation** | **Blind rater** | **Physical examination** | **Randomization of exercises** | **Familiarization of exercises** | **Standardization of exercise technique** | **Randomization of MVIC_s_** | **Appropriate normalization** | **Appropriate statistical tests** |
| Ratanapinunchai et al. (2022) | **No** | **Yes** | **Yes** | **No** | **No** | **Yes** | **No** | **No** | **No** | **Yes** | **Yes** |
| De Faria et al.  (2021) | **No** | **Yes** | **Yes** | **No** | **Yes** | **Yes** | **No** | **Yes** | **Yes** | **Yes** | **Yes** |
| Patselas et al.  (2021) | **No** | **Yes** | **Yes** | **No** | **No** | **Yes** | **No** | **Yes** | **Yes** | **Yes** | **Yes** |
| Ferreira et al.  (2020) | **No** | **Yes** | **Yes** | **No** | **Yes** | **No** | **Yes** | **Yes** | **No** | **Yes** | **Yes** |
| De Araujo et al.  (2020) | **No** | **Yes** | **Yes** | **No** | **Yes** | **No** | **Yes** | **Yes** | **Yes** | **Yes** | **Yes** |
| Youdas et al.  (2020) | **Yes** | **Yes** | **Yes** | **No** | **Yes** | **Yes** | **Yes** | **Yes** | **No** | **Yes** | **Yes** |
| Kim and Yoo  (2019) | **No** | **Yes** | **Yes** | **No** | **No** | **Yes** | **No** | **Yes** | **No** | **Yes** | **Yes** |
| De Araujo et al.  (2018) | **No** | **Yes** | **Yes** | **No** | **Yes** | **Yes** | **Yes** | **Yes** | **Yes** | **Yes** | **Yes** |
| Harris et al.  (2017) | **Yes** | **Yes** | **Yes** | **No** | **No** | **Yes** | **No** | **No** | **Yes** | **Yes** | **Yes** |
| Torres et al.  (2017) | **No** | **Yes** | **Yes** | **No** | **No** | **Yes** | **Yes** | **Yes** | **No** | **Yes** | **Yes** |
| Horsak et al.  (2017) | **No** | **Yes** | **Yes** | **No** | **No** | **Yes** | **Yes** | **Yes** | **Yes** | **Yes** | **Yes** |
| Gioftsos et al.  (2016) | **No** | **Yes** | **Yes** | **No** | **No** | **Yes** | **Yes** | **Yes** | **No** | **Yes** | **Yes** |
| Kim et al.  (2016) | **No** | **Yes** | **Yes** | **No** | **No** | **Yes** | **No** | **No** | **No** | **Yes** | **No** |
| Lee et al.  (2015) | **No** | **Yes** | **Yes** | **No** | **No** | **Yes** | **No** | **No** | **No** | **Yes** | **No** |
| Herrington et al.  (2015) | **Yes** | **Yes** | **Yes** | **No** | **No** | **No** | **No** | **No** | **No** | **Yes** | **Yes** |
| Borreani et al. ^(a)^  (2015) | **No** | **Yes** | **Yes** | **No** | **No** | **Yes** | **Yes** | **Yes** | **No** | **Yes** | **Yes** |
| Borreani et al. ^(b)^  (2015) | **No** | **Yes** | **Yes** | **No** | **No** | **Yes** | **Yes** | **Yes** | **No** | **Yes** | **Yes** |
| De Mey et al.  (2014) | **Yes** | **Yes** | **Yes** | **No** | **No** | **Yes** | **Yes** | **Yes** | **No** | **Yes** | **Yes** |
| Calatayud et al. ^(a)^ (2014) | **No** | **Yes** | **Yes** | **No** | **No** | **Yes** | **Yes** | **Yes** | **No** | **Yes** | **Yes** |
| Calatayud et al. ^(b)^  (2014) | **No** | **Yes** | **Yes** | **No** | **No** | **Yes** | **Yes** | **Yes** | **No** | **Yes** | **Yes** |
| Calatayud et al. ^(c)^  (2014) | **No** | **Yes** | **Yes** | **No** | **No** | **Yes** | **Yes** | **Yes** | **No** | **Yes** | **Yes** |
| McGill et al.  (2014) | **No** | **Yes** | **Yes** | **No** | **No** | **Yes** | **Yes** | **Yes** | **No** | **Yes** | **Yes** |
| Kim et al.  (2014) | **No** | **Yes** | **Yes** | **No** | **Yes** | **Yes** | **Yes** | **No** | **No** | **Yes** | **Yes** |
| Yoo  (2013) | **No** | **Yes** | **Yes** | **No** | **No** | **No** | **No** | **No** | **No** | **Yes** | **Yes** |
| Park et al. ^(a)^  (2013) | **No** | **Yes** | **Yes** | **No** | **No** | **No** | **No** | **Yes** | **No** | **Yes** | **Yes** |
| Park et al. ^(b)^  (2013) | **No** | **Yes** | **Yes** | **No** | **No** | **Yes** | **No** | **Yes** | **No** | **Yes** | **Yes** |
| Yoon and Lee  (2013) | **No** | **Yes** | **Yes** | **No** | **No** | **No** | **Yes** | **No** | **No** | **Yes** | **Yes** |
| Seo et al.  (2013) | **No** | **Yes** | **Yes** | **No** | **Yes** | **Yes** | **Yes** | **No** | **No** | **Yes** | **Yes** |
| Lee et al.  (2013) | **No** | **Yes** | **Yes** | **No** | **No** | **Yes** | **Yes** | **No** | **No** | **Yes** | **Yes** |
| Park and Yoo  (2013) | **No** | **Yes** | **Yes** | **No** | **No** | **Yes** | **Yes** | **Yes** | **No** | **Yes** | **Yes** |
| Kim et al.  (2012) | **No** | **Yes** | **Yes** | **No** | **No** | **Yes** | **No** | **No** | **No** | **Yes** | **Yes** |
| Park and Yoo  (2011) | **No** | **Yes** | **Yes** | **No** | **No** | **Yes** | **Yes** | **Yes** | **No** | **Yes** | **Yes** |
| Tucker et al.  (2011) | **Yes** | **Yes** | **Yes** | **No** | **No** | **Yes** | **Yes** | **Yes** | **No** | **Yes** | **Yes** |
| Maenhout et al.  (2010) | **Yes** | **Yes** | **Yes** | **No** | **No** | **Yes** | **No** | **Yes** | **No** | **Yes** | **Yes** |
| Sandhu et al.  (2008) | **No** | **Yes** | **Yes** | **No** | **No** | **Yes** | **Yes** | **No** | **No** | **Yes** | **Yes** |
| Lehman et al.  (2008) | **No** | **Yes** | **Yes** | **No** | **No** | **Yes** | **No** | **No** | **No** | **Yes** | **Yes** |
| Tucker et al.  (2008) | **Yes** | **Yes** | **Yes** | **No** | **No** | **Yes** | **Yes** | **Yes** | **No** | **Yes** | **Yes** |
| Lear and Gross  (1998) | **Yes** | **Yes** | **Yes** | **No** | **No** | **Yes** | **Yes** | **No** | **No** | **Yes** | **Yes** |
